# Complete mitochondrial genome of *Sinogastromyzon szechuanensis* (Teleostei, Cypriniformes, Balitoridae) obtained using next-generation sequencing

**DOI:** 10.1080/23802359.2020.1846471

**Published:** 2021-01-12

**Authors:** Wei Li, Xiaggojiang Chen, Mengmeng Zheng, Yin Wang, Xingchen Zhou, Haiyang Li, Guolong Zhou, Bichun Li

**Affiliations:** a College of Animal Science and Technology, Yangzhou University, Yangzhou, Jiangsu, China; b Department of Fishery Sciences and Technology, Jiangsu Agri-animal Husbandry Vocational College, Taizhou, Jiangsu, China; c Jiangyan County Agricultural and Rural Bureau, Taizhou, Jiangsu, China

**Keywords:** Mitochondrial genome, *Sinogastromyzon szechuanensis*, next-generation sequencing

## Abstract

The complete mitogenome sequence of *Sinogastromyzon szechuanensis* was obtained using next generation sequencing and confirmed via overlap-PCR method. The genome was 16,565 bp in length and consisted of 13 protein-coding genes (PCGs), 2 ribosomal RNA genes, 23 transfer RNA genes and 1 control region. The overall nucleotide composition of heavy strand was 30.38% A (5033), 16.62% G (2753), 25.25% T (4182) and 27.75 (4597), with a slight A–T skew (55.63%), which is most obvious in the D-loop and most transfer RNA genes. Mitochondrial genome analyses based on ML analyses yielded identical phylogenetic trees.

*Sinogastromyzon szechuanensis Fang* (Ding 1994), belonging to Balitoridae of Cypriniform, is a small freshwater and endemic to the upper Yangtze River, China. Being different from other Cypriniform fish, *S. szechuanensis* is of flattened body and capable of jumping among the stones of waterfall or mountain streams (Jin-Ming et al. 2011; Wu et al. 2013). They are of high economic value for their good taste. Unfortunately, they were in danger for their high price and shrunken habitat. There were only a few documents or researches focus on them. It will be a tremendous priority to take action to protect and use them carefully.

The *S. szechuanensis* were collected from *Neijiang*, *Sichuan*, *China* (29°36′41.93″N, 105°01′47.67″E) and stored at Aquatic Science and Technology Institution Herbarium (Accession number: SC20180913HXQ02). Total genomic DNA of liver was extracted using Magnetic Animal Tissue Genomic DNA kit of Tiangen Biotech (Beijing) Co., Ltd (Yu et al. 2019). The genomic DNA (DNA label: *S. szechuanensis* 001) were stored in an ultralow refrigerator (–70 °C) of the Herbarium.

A genomic DNA library was established and sequenced to assembly the whole mitochondrial genome using next-generation sequencing method (Illumina HiSeq platform) (Asem et al. 2018). Quality check for sequencing data was done by FastQC and the fragments sequences were assembled and mapped using SPAdes (Bankevich et al. 2012). Thirty-five pairs of PCR primers were designed to amplify the whole mitogenome sequence based on the assembled DNA sequences to affirm the assembled results.

The complete mitogenome of *S. szechuanensis* is 16,565 bp in length (NCBI accession ID: MN241814) and of conserved structural organization, which consisted of 13 protein-coding genes (PCGs), 2 ribosomal RNA genes, 23 transfer RNA genes and 1 control region displacement loop (D-loop). The overall nucleotide composition of heavy strand was 30.38% A (5033), 16.62% G (2 753), 25.25% T (4 182) and 27.75 (4 597), with a slight A–T skew (55.63%), which is most obvious in the D-loop and most transfer RNA genes. Except for seven RNAs (*tRNA-Ala, tRNA-Asn, tRNA-Cys, tRNA-Tyr, tRNA-Ser, tRNA-Glu* and *tRNA-Pro*) and one protein (*ND6*), which were encoded on the L-strand, most elements were encoded on the heavy strand (H-strand). 12 s rRNA (944 bp) and 16 s rRNA (1 508 bp) were separated by a gap of 151 bp, while tRNA-Val was encoded within the gap.

The maximum-likelihood (ML) phylogenetic (Zhu et al. 2018) tree was constructed with MEGA 6.0 program based on 13 complete mitochondrial genome sequences (Asem et al. 2018) (Figure 1). The results showed a close relationship among *S. szechuanensis, S. hsiashiensis* (KY352771), *S. sichangensis* (NC_024534), *S. puliensis* (NC_011922), *S. tonkinensis* (KY352773) and a relative loose relationship between *S. szechuanensis* and *Danio retio* (KM244705). In conclusion, this complete mitochondrial genome would establish a solid foundation for future population geography and conservation genetic studies of *S. szechuanensis*.

**Figure 1.**
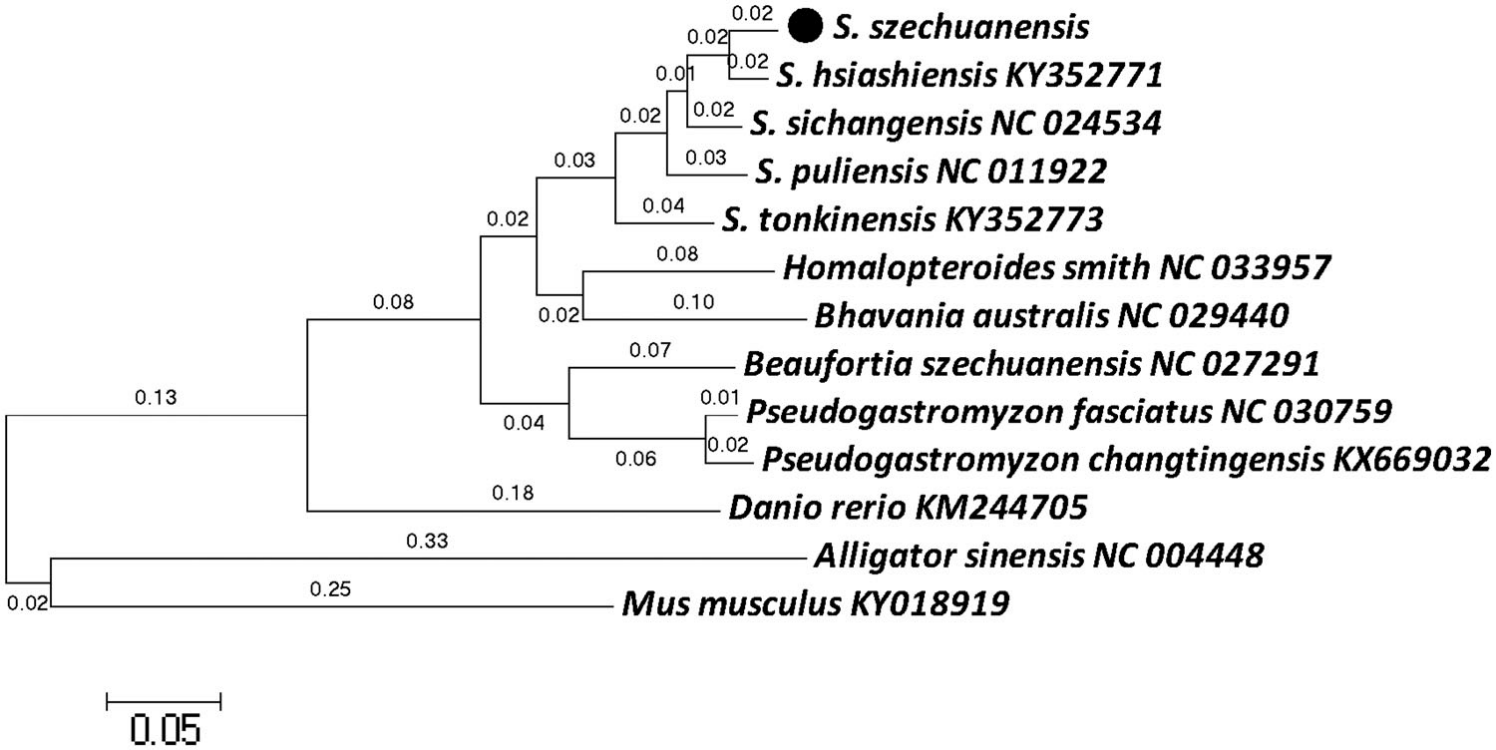
Phylogenetic tree showing the relationship among *S. szechuanensis*, 4 species of *Sinogastromyzon*, 5 other species of Balitoridae and 3 outgroup samplings bases on maximum-likelihood (ML) approach.

## Data Availability

Mitogenome data supporting this study are openly available in GenBank at: https://www.ncbi.nlm.nih.gov/nuccore/MN241814.1 Associated BioProject, SRA, and BioSample accession numbers are https://www.ncbi.nlm.nih.gov/bioproject/ PRJNA667134, https://www.ncbi.nlm.nih.gov/sra/ SRR12767438, and SAMN16356296, respectively.
